# The Prevalence of Pronator Teres among Patients with Carpal Tunnel Syndrome: Cross-sectional Study

**Published:** 2016-09

**Authors:** Mahsa Asheghan, Mohammad Taghi Hollisaz, Abbas Shahabi Aghdam, Amidoddin Khatibiaghda

**Affiliations:** Department of Physical Medicine and Rehabilitation, School of Medicine, Baqiytallah University of Medical Sciences, Iran

**Keywords:** Median Nerve, Carpal Tunnel Syndrome, Pronator Teres Syndrome, Electrodiagnostic Study

## Abstract

The aim of conducting this study was to determine the prevalence of PTS among patients with carpal tunnel syndrome. The study was conducted from March 2014 to April 2015 in the EDX ward and clinic of physical medicine and rehabilitation at the university hospital; Baqiytallah, a large referral practice and research center in Tehran. We included patients with clinical symptoms and signs of CTS. Clinical assessments were aimed to the diagnosis of CTS and PTS. At the next stage, ultrasound study was performed for the participants with suspected CTS. Sample size calculations were based on the formula:

$$\text{N}=4[\text{pq}/{\text{w}}^{2}]{\text{z}}_{1-\alpha /2}^{2}$$.

Results showed that 13 (8.8%) patients presented electrodiagnostic, and 27 (18.2%) had clinical manifestations of pronator teres syndrome of which, 17 showed ultrasonic signs of the syndrome. In addition, 2, 7, and 8 out of the 17 patients had mild, moderate, and sever carpal tunnel syndrome, respectively. Age was not significantly different between the patients with, and without pronator teres syndrome (*p*-value=0.179). Nine participants with pronator teres syndrome were male and there was a significant difference concerning sex (*p*-value=0.013). There was a good agreement between electrodiagnostic and ultrasound findings (Cohen’s kappa coefficient=0.71, *p*-value<0.0001). Taken together, pronator teres syndrome should be considered as a possibility among patients with carpal tunnel syndrome especially in sever forms. Both electrodiagnostic and sonographic studies are efficient for diagnosing pronator teres syndrome. Men are more prone to develop pronator teres syndrome.

## INTRODUCTION

Symptomatic compression neuropathy of the median nerve is a frequently encountered clinical entity (1, 2). The most common form of the median nerve compression is carpal tunnel syndrome (CTS) (3). Patients with CTS present frequently with numbness, tingling, hand and arm pain and motor weakness at the level of the wrist (1, 2).

Taking an accurate patient history, performing physical examination, and obtaining electrodiagnostic (EDX) tests have been recommended in the workup of patients with CTS (2). Splints, local injection of corticosteroids, and surgical decompression have been used as the treatment modalities for CTS.

However, in some situations therapeutic measures do not culminate in favorable outcome. Lack of optimal relief of symptoms following carpal tunnel surgery may be due to the failure to identify the second site of compression (4). In certain cases, clinical manifestations are indicative of median nerve compression in locations proximal to the carpal tunnel (1, 3, 5-11). Yet, because of overlapping symptoms, CTS is sometimes diagnosed and a more proximal site of compression is missed (4, 12-14). In addition, there may be a coexisting pathology along the median nerve course together with CTS (15). Anatomical knowledge, rigorous clinical evaluations and EDX studies have been recommended to avoid pitfalls in the differential diagnosis of CTS (7, 14, 16-21). Accurate diagnosis will shorten morbidity time and prevent exposure of patients to additional operations (4).

Proximal to carpal tunnel, the median nerve traverses between the heads of the pronator teres and then enters deep to the tendinous arch connecting the radial and humeral heads of the flexor digitorum superficialis muscle. Beside of compression at the carpal tunnel, the median nerve may be compressed between the heads of the pronator teres muscle (3, 16). Patients complain of pain, numbness or paresthesia over the anterior forearm and lateral 3.5 digits that aggravate with forced pronation. Nerve conduction velocity (NCV) or amplitude of the median nerve may decrease in the forearm, but distal sensory and motor latencies are normal except when there is an associated CTS (11, 12). However, the exact pattern of these abnormalities is uncertain. Existing evidence regarding pronator teres syndrome (PTS) is inadequate, and its prevalence among patients with CTS has not been properly estimated.

The aim of conducting this study was to determine the prevalence of PTS among patients with CTS. We assessed EDX data, clinical manifestations, and demographic characteristics in patients with the two syndromes, and reported the relationships of EDX results and clinical manifestations with sonographic findings in patients with PTS. We hypothesize that decrease in the NCV of the median nerve in CTS might be due to retrograde damage as well as concurrent pathology proximal to the carpal tunnel.

## METHODOLOGY

### Ethical considerations

The study was conducted in accordance with the Declaration of Helsinki, and the research protocol was approved by the ethics committee of Baqiatallah University of Medical Sciences. All patients signed written consents and they were referred for appropriate treatment, if needed. Patients were informed that they were free to withdraw from the study at any time.

### Design

We performed a cross-sectional study. The study was conducted from March 2014 to April 2015 in the EDX ward and clinic of physical medicine and rehabilitation at the university hospital; Baqiytallah, a large referral practice and research center in Tehran.

### Participants

We recruited patients with the manifestations of CTS who came to the clinic, or they were referred by physicians requiring assistance with workup of the syndrome. At the first visit medical histories were completed, physical examinations were performed and further investigations were ordered, if needed. Then, the patients were screened for eligibility and were listed for EDX studies. From the eligible individuals we randomly selected our analytic sample according to a predetermined sample size. Clinical examinations were performed by a senior resident, and EDX studies by a physiatrist. The study was carried out under the supervision of a professor in physical medicine and rehabilitation.

### Eligibility criteria

We included patients with clinical symptoms and signs of CTS. There was no restriction for sex. Exclusion criteria were concurrent neuropathy, history of trauma to the upper limb or previous surgery, cervical radiculopathy, age more than 65 years (because of senile reduction in NCV), and also manifestations of osteoarthritis, rheumatoid arthritis, amyloidosis, and gout. We also excluded patients with possible systemic causes of CTS such as diabetes, hypothyroidism, renal failure, and autoimmune diseases, and if they were undergoing haemodialysis. Participants with body mass index more than 30 kg/m^2^ were excluded, too. Individuals who could not tolerate the EDX study did not entered to the study.

### Protocols and procedures

**Clinical evaluations.** Clinical assessments were aimed to the diagnosis of CTS and PTS. A research assistant recorded history and demographic data through interview and a self-administered questionnaire. The amount of physical activity, lifestyle, and comorbid conditions were asked and the presence of numbness, tingling, and pain recorded. Visual analog scores (VAS) were used for measuring subjective severity of pain. Participants recorded their pain perception by corresponding it to a 100-mm line ranging from 0 (no pain) to 9 (worst pain). Characteristics of symptoms were noted including location, duration, severity, and worsening at night.

Physical examinations included: observation of deformity, assessment of pain with pronation against resistance, the presence of tenderness on the pronator teres muscle, sensory and motor examinations, muscle testing for atrophy, and Tinel’s test. Radiographs of the wrist were obtained if needed. In addition, any previous consultation with a physician and EDX study or any current treatment was investigated carefully. For equivocal situations, a consensus committee decided for the diagnosis.

**Electrodiagnostic studies.** Participants were stratified by CTS severity as mild: prolonged either sensory or motor distal latency (>3.5 and > 4.5 ms, respectively); moderate: prolonged both sensory and motor distal latencies; severe: when sensory or motor (or both) distal latencies were not measurable. For the patients with severe CTS or in cases of absent latency, AIN (anterior interosseous nerve) conduction studies of the ulnar and radial nerves were used to differentiate CTS with peripheral neuropathies or PTS. We performed EMG (electromyography) of 6 upper limb muscles innervated by cervical nerves and the paraspinal muscles. Beside of routine EDX studies for the diagnosis of CTS, the median NCS along the forearm and in the elbow, AIN CAMP latency, comparison of CAMP amplitudes at the two sides, AIN and EMG of the muscles innervated by the median nerve were carried out. The skin temperature of the forearm and wrist were kept at 32-33°C during all measurements.

**Ultrasound studies.** At the next stage, ultrasound study was performed for the participants with suspected CTS. We investigated the position of the median nerve between the two heads of the pronator teres dynamically during pronation of the forearm, and measured the cross-section of the nerve before, after, and at the site of crossing the pronator teres, statically. Any noticeable reduction in cross-sectional diameter along with decrease in the nerve mobility throughout flexion, supination, and pronation was considered as the criterion for entrapment of the median nerve. In addition, any sign of fusiform enlargement and flattening of the nerve were investigated. The sonography (Mylab 25 gold, Esoate, Italy) was performed with 10 to 18 mega Hz, high resolution probe, and musculoskeletal and nerve protocol.

**Outcome measures.** The primary outcome measure was the prevalence of PTS among patients with CTS. We also estimated the prevalence of concurrent CTS and PTS within the strata of CTS severity; and assessed the relationship between clinical, ultrasound, and EDX findings. In addition, we investigated the prevalence of the syndromes with respect to age and sex.

**Sample size calculations.** Sample size calculations were based on the formula:

$$\text{N}=4[\text{pq}/{\text{w}}^{2}]{\text{z}}_{1-\alpha /2}^{2}$$,

where *p* is the anticipation of the prevalence of CTS; *q* = 1- *p*; *ω* is the planned width of 95% CI for the estimation of the prevalence, *α* = 0.05, and *z_0·975_* = 1.9600. For the anticipated prevalence of 10%, we at least needed to include 138 participants to provide the planned width ω = 0.1 of 95% CI for the estimation of the prevalence. We added a more 10 participants and included 148 patients in our study.

### Statistical analyses

For each participant, we analyzed data from the hand with more sever CTS. If both hands were the same in the intensity of the syndrome, one hand was selected randomly. Data was presented as mean (standard deviation), for continuous variables, and as numbers and proportions for categorical variables. Categorical variables were compared by means of the chi-square or Fisher’s exact test, as appropriate. Data was analyzed with a statistical soft ware package (SPSS for Windows, version 13, SPSS, Inc., Chicago, IL, USA). A *p*-value of less than 0.05 was considered significant, and the power of statistical tests was set at 80%.

## RESULTS

Our sample was composed of 148 participants (148 hands). Mean age (std) was 51.4 (7.6) with the range of 21 to 63 years. Of these patients, 107 (72.3%) were female. Participants had various occupations from low to high physical levels of activity. We had 81 (54.7%) right and 67 (45.3%) left CTS. Regarding the severity of CTS, 57 (38.5%) had mild, 55 (37.2%) had moderate, and 36 (24.3%) had sever CTS. Of the 148 participants, 13 (8.8%) presented EDX manifestations of PTS, and 27 (18.2%) had clinical PTS.

Of the patients with clinical PTS, 17 (11.5% of the sample) showed ultrasonic signs of compression of the median nerve between the two heads of the pronator teres muscle. Of these 17 participants, 10 presented the involvement of the median nerve at the right side. In addition, 2, 7, and 8 out of these 17 patients had mild, moderate, and sever CTS, respectively [Pearson’s chi-squared (2) = 7.32, *p*-value = 0.021]. We also assessed AIN latency for the 17 participants. In 8 patients, the latency was > 8 ms and in 10, difference of the latencies between the two sides was > 70 ms. In 12 individuals, either AIN latency > 8 ms or the discrepancy > 20% (0.7 ms) between the two sides was detected.

We had 13 patients for which the diagnosis of PTS was confirmed with EDX studies. Three patients showed decreased median forearm velocity in their hands. Eight out of these 13 participants had either abnormal conduction velocity or abnormal amplitude. Needle exam showed an abnormality of at least one median innervated muscle, abductor pollicis brevis, flexor carpi radialis, or pronator teres, in 10 of the 13 patients. All the 13 participants with EDX-proven PTS showed positive ultrasound findings for the syndrome while in 4 patients with clinical PTS ultrasound was not productive. Of these 13 individuals, 8 had AIN latency > 4ms, 9 had right-versus-left discrepancy of the latencies > 20% (0.7 ms), and 11 had either or both the abnormalities was seen. There was no statistically significant difference between EDX- and ultrasonography-proven PTS in term of AIN latency abnormalities (*p*-values > 0.05).

Mean (std) age for the patients with PTS was 53.7 (6.5) years. There was not statistically significant difference between the patients with, and without PTS with respect to age [t-test (146) = 1.350, mean difference = 2.6 years, *p*-value = 0.179]. Nine participants with PTS were male and there was a statistically significant difference concerning sex [Pearson’s chi-squared (1) = 6.108, *p*-value = 0.013] among the participants with PTS. Table 1 shows the relationship between clinical and EDX studies for the diagnosis of PTS. Considering clinical diagnosis of PTS as the standard, sensitivity for EDX was 48% (95% CI = 40% to 56%) and specificity was 100%. Table 2 demonstrates the relationship between EDX and ultrasound findings among patients who underwent ultrasonography. There was a good agreement between EDX and ultrasound findings (Cohen’s kappa coefficient = 0.71, std= 0.13, proportion in agreement = 0.85, *p*-value < 0.0001).

**Table 1:** Relationship between clinical and EDX studies for the diagnosis of PTS. Figures are numbers (percentages)

		Clinical PTS		
		positive	negative	total

EDX	positive	13 (8.8)	0 (0.0)	13 (8.8)
	negative	14 (9.4)	121 (81.8)	135 (91.2)
	total	27 (18.2)	121 (81.8)	148 (100)

**Table 2:** Relationship between EDX and ultrasound findings for PTS. Figures are numbers (percentages)

		EDX		
		positive	negative	total

Ultrasound	positive	13 (48.1)	4 (14.8)	17 (63.0)
	negative	0 (0.0)	10 (37.0)	10 (37.0)
	total	13 (48.1)	14 (51.9)	27 (100)

## DISCUSSION

We tried to determine the prevalence of PTS among patients with CTS. We compared clinical findings with the results of EDX studies, and performed sonographic assessments for the patients with the two syndromes. Our study showed that PTS should be considered as a possibility among patients with CTS, and that the association is stronger for sever CTS. We found that both EDX and sonographic studies are efficient means for diagnosing PTS, and their results are well correlated. Especially, it seems that EDX studies are highly specific for the diagnosis of pronator teres syndrome. Age is not a significant predictor of concurrent PTS. In contrast, sex is significant as men were more prone to develop PTS in our sample.

To our knowledge, there is no recent study on the prevalence of PTS in patients with CTS. In our study, statistical analyses were straightforward and missing data were trivial. The size of the sample was sufficiently large. We used standard and easy to follow protocols, and our research team and assessors were highly trained and attempted to follow the protocols strictly.

Overall, our results are consistent with previous findings reported in the literature. Researchers have recently studied cross-section areas of median nerve and its correlation with the nerve conduction study, using high-resolution ultrasonography (22). They included a sample of 212 CTS and 50 asymptomatic hands in their study. They found that the cross-sectional area has positive correlations with nerve conduction study and the severity and duration of symptoms. Also they declared that combined nerve conduction and ultrasonography studies may yield a higher positive rate than nerve conduction study alone for diagnosing CTS. Our study also showed that EDX and sonographic findings are confirmatory for the diagnosis of PTS.

In another study, researchers evaluated the relationship between the results of nerve conduction study and sonographic findings in patients with type 2 diabetes mellitus (23). They enrolled 30 patients and 32 healthy volunteers and assessed cross-sectional area of the median nerve at the carpal tunnel proximal to the wrist. They found a significant increase in the cross-sectional and hypoechoic area of the nerve in diabetic patients compared with controls (*P*<0.05). They reported that cross-sectional area was negatively correlated with reduced motor nerve conduction velocity and delayed latency, and concluded that sonographic examinations are useful for the diagnosis of diabetic neuropathy. Other more previous studies have also highlighted these results (24).

Some researchers focused on slowing motor conduction velocity of the forearm median nerve in patients with CTS, when either focal conduction abnormality over wrist or retrograde conduction slowing is present. They also aimed to determine whether the slowing is correlated to severity of compression or not. Fifty patients with nerve conduction study proven CTS, and 100 participants as controls were included. They concluded that the retrograde conduction slowing occurs among patients with CTS prominantly in those with abnormal electromyography, and mildly in those with only demyelination. They rejected the conventional belief that nerve function changes only distal to injured sites (25).

In a retrospective study, records of 83 limbs in seventy-two patients with PTS were reviewed (12). The reviewed items included demographic, clinical, and EDX data, as well as treatment modalities. Slowing of median forearm velocity was seen in 25 limbs (30%), and in 54 (65%) median sensory findings of either abnormal conduction velocity or abnormal amplitude was detected. In our study, 13 (48%) of 27 patients with PTS showed slowing of forearm median conduction velocity. The discrepancy may be due to different sample sizes and the inconsistent designs of the two studies. Meanwhile, the two researches roughly suggest the same clinical implication.

In a more previous research, 39 limbs in 36 patients who underwent surgical decompression of the median nerve in the proximal forearm were evaluated. 24 Elbow to wrist nerve conduction velocity was assessed in 37 limbs and was abnormal in 12 (32%). Although this figure is close to our estimation, but the two studies are basically different in design. In another study, EDX and clinical findings in 17 patients with proximal median neuropathy were reviewed (25)> The causes of neuropathy were trauma in 5, pronator teres overuse in 3, postinfectious in 2, secondary to a congenital lesion in 1, and undetermined in 6 patients. In 14 patients the neuropathy was present at or proximal to the pronator teres muscle and in 3 at the anterior interosseous portion of the nerve. It was reported that EDX results were more definitive than clinical findings.

## CONCLUSION

Overall, we did not find any similar study the results of which would be compared to ours. Our results have two important implications for the workup of patients with CTS in general practice. Because of overlapping symptoms or the possibility of coexisting pathology, patients with CTS have to be carefully evaluated for the presence of PTS. In addition, clinical diagnosis of PTS should be confirmed with ultrasonography or EDX studies, especially when the patient is a candidate for surgery. Our findings can be considered as a basis for further research particularly for calculations of sample size.
